# Quality control of elbow joint radiography using a YOLOv8-based artificial intelligence technology

**DOI:** 10.1186/s41747-024-00504-7

**Published:** 2024-09-20

**Authors:** Qi Lai, Weijuan Chen, Xuan Ding, Xin Huang, Wenli Jiang, Lingjing Zhang, Jinhua Chen, Dajing Guo, Zhiming Zhou, Tian-wu Chen

**Affiliations:** Department of Radiology, The Second Affiliated Hospital of Chongqing Medical University, Yuzhong, Chongqing, China

**Keywords:** Artificial intelligence, Deep learning, Elbow joint, Quality control, Radiography

## Abstract

**Background:**

To explore an artificial intelligence (AI) technology employing YOLOv8 for quality control (QC) on elbow joint radiographs.

**Methods:**

From January 2022 to August 2023, 2643 consecutive elbow radiographs were collected and randomly assigned to the training, validation, and test sets in a 6:2:2 ratio. We proposed the anteroposterior (AP) and lateral (LAT) models to identify target detection boxes and key points on elbow radiographs using YOLOv8. These identifications were transformed into five quality standards: (1) AP elbow positioning coordinates (X_A_ and Y_A_); (2) olecranon fossa positioning distance parameters (S_17_ and S_27_); (3) key points of joint space (Y_3_, Y_4_, Y_5_ and Y_6_); (4) LAT elbow positioning coordinates (X_2_ and Y_2_); and (5) flexion angle. Models were trained and validated using 2,120 radiographs. A test set of 523 radiographs was used for assessing the agreement between AI and physician and to evaluate clinical efficiency of models.

**Results:**

The AP and LAT models demonstrated high precision, recall, and mean average precision for identifying boxes and points. AI and physicians showed high intraclass correlation coefficient (ICC) in evaluating: AP coordinates X_A_ (0.987) and Y_A_ (0.991); olecranon fossa parameters S_17_ (0.964) and S_27_ (0.951); key points Y_3_ (0.998), Y_4_ (0.997), Y_5_ (0.998) and Y_6_ (0.959); LAT coordinates X_2_ (0.994) and Y_2_ (0.986); and flexion angle (0.865). Compared to manual methods, using AI, QC time was reduced by 43% for AP images and 45% for LAT images (*p* < 0.001).

**Conclusion:**

YOLOv8-based AI technology is feasible for QC of elbow radiography with high performance.

**Relevance statement:**

This study proposed and validated a YOLOv8-based AI model for automated quality control in elbow radiography, obtaining high efficiency in clinical settings.

**Key Points:**

- QC of elbow joint radiography is important for detecting diseases.
- Models based on YOLOv8 are proposed and perform well in image QC.
- Models offer objective and efficient solutions for QC in elbow joint radiographs.

**Graphical Abstract:**

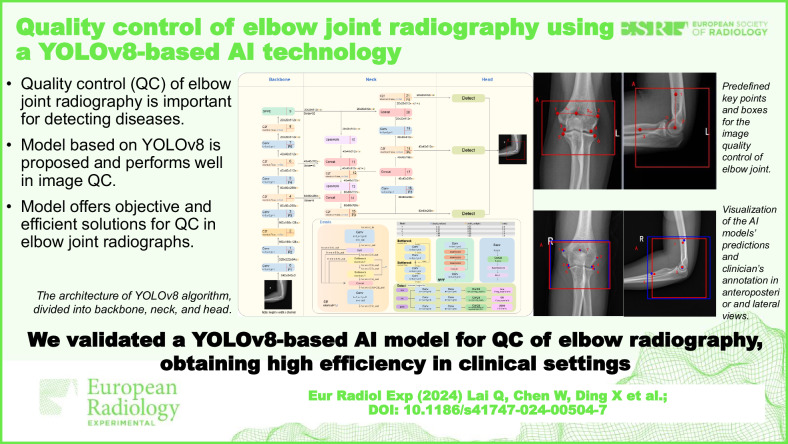

## Background

The elbow is a joint at risk of injuries from various sources, including trauma, routine activities, aging, overuse, and diseases such as rheumatoid arthritis, osteoarthritis, infections, and tumors [1]. Despite advanced orthopedic treatments, outcomes for elbow injuries often fall short of optimal due to its intricate anatomy. This complexity necessitates regular radiological evaluations. Radiography remains a vital diagnostic resource, even with the development of more advanced imaging techniques [2, 3]. Anteroposterior (AP) and lateral (LAT) radiographs are extensively used to determine whether patients have disorders such as fractures, dislocations, and arthritis [4]. High-quality elbow radiographs are critical for the precise depiction. Poor-quality radiographs, largely due to incorrect patient positioning or radiographer errors, can compromise clinical decision-making. The current rejection rate for elbow radiographs is approximately 9.3% [5].

Quality control (QC) in radiography is crucial for diagnosing elbow diseases, involving key metrics like signal-to-noise ratio, sharpness, and contrast. Although many studies assess x-ray quality based on factors such as sharpness and contrast [6, 7], these are not the primary focus in digital radiography. A crucial QC factor is the correct alignment of the irradiated body part in relation to the x-ray equipment: an x-ray image, regardless of its sharpness and contrast, is ineffective if it fails to accurately display anatomical structures due to misalignment. Therefore, for clinical requirements, the focus for image QC should be on positioning.

Elbow joint radiographs must meet the following specific standards as follows. For AP elbow joint radiographs, the following conditions should be met [4]: (1) the distal humerus, proximal ulna and radius, and their joint spaces are centered in the image; (2) the elbow joint surfaces are tangentially aligned, sharply defined, with the coronoid fossa slightly ulnar to the center of the medial and lateral epicondyles of the humerus; and (3) the bone trabeculae and surrounding soft tissues of the elbow joint are clearly visible. For LAT elbow joint radiographs, the standards include: (1) the distal humerus and proximal ulna and radius form a 90° angle, with the ulnohumeral joint space clearly and sharply displayed [8]; (2) the lateral epicondyle of the humerus overlaps, forming a circular projection; and (3) bone trabeculae of the elbow joint is distinct, with clear delineation of surrounding soft tissues. Currently, AP and LAT elbow radiograph QC primarily depend on visual evaluation, potentially influenced by radiologists’ subjective factors. This underscores the need for automated real-time QC tools in x-ray radiography to determine if retakes are necessary, thereby reducing medical errors and improving patient satisfaction.

The rapid advancement in artificial intelligence (AI), particularly deep learning, shows significant potential in medical image QC. Deep learning models, trained on extensive data, can match or even exceed human experts in medical image QC. Various studies focus on the development of deep learning models for QC in chest x-rays, knee radiographs, ankle radiographs, and lumbar spine x-rays, often matching or exceeding human experts [9–12]. To our knowledge, there are no AI-based methods for QC assessment of elbow joint radiographs.

For AI-based medical image QC, real-time performance is essential. YOLO, short for “You Only Look Once”, is acclaimed for its fast real-time detection and diverse applications. This algorithm’s primary advantage is its ability to rapidly and accurately detect objects’ class and location in real-time, aligning well with the needs of medical image QC [13, 14]. In April 2023, Ultralytics (Frederick, Maryland, USA) released the latest version of the YOLO algorithm, YOLOv8, which further enhances detection accuracy while maintaining high speed [15–18].

In this study, we aimed to explore the feasibility of applying the latest AI technology based on the YOLO model for automated QC of elbow joint radiographs. The goal was to determine if our model aligns with professional standards, offering a reliable AI solution for elbow radiograph QC.

## Methods

This retrospective study was granted ethical approval by our university hospital (No. (2024)681). Retrospective imaging data were utilized, and all individual data were anonymized, obviating the need for personal written informed consent.

### Data collection

Drawing upon the methodologies outlined in the pertinent literature [10], the data collection in this study adhered to strict inclusion and exclusion criteria. The inclusion criteria were: (1) subjects were adults aged over 18 years; (2) subjects underwent radiography of the elbow; and (3) the obtained elbow joint radiographs complied with relevant radiological standard guidelines. The exclusion criteria included: (1) elbow joint radiographs were not in AP or LAT view; (2) elbow joint radiographs were blurry or partially obscured; (3) elbow joint radiographs showed fractures, foreign objects, or postoperative changes; or (4) elbow joint radiographs were obtained under incorrect shooting conditions.

Based on the above inclusion and exclusion criteria, we conducted a retrospective analysis of 2,643 elbow joint radiographs collected from January 2022 to August 2023 at our hospital. These images were derived from 1,679 consecutive patients, including both outpatients and inpatients. Among these radiographs, 1,317 were AP views, and 1,326 were LAT views. To construct a deep learning model, we randomly selected 1,598 images from 1,038 patients as the training set (including 800 AP views and 798 LAT views), 522 images from 317 patients as the validation set (including 257 AP views and 265 LAT views), and the remaining 523 images from 324 patients (including 260 AP views and 263 LAT views) as the test set. The distribution of these datasets was approximately in a 6:2:2 ratio.

**Fig. 1:**
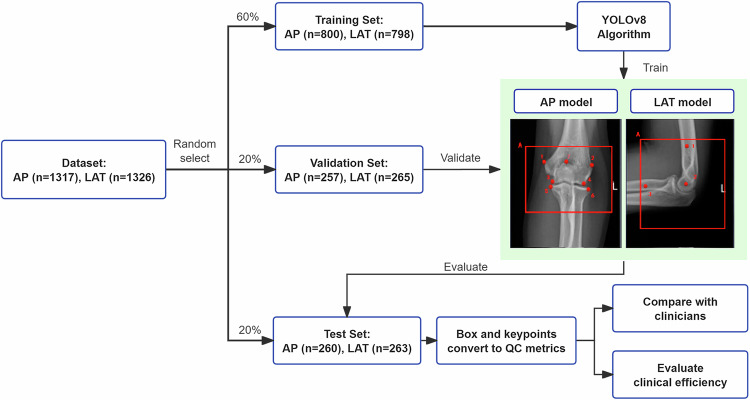
Flowchart of the model training, validation and test on the dataset. AP, Anteroposterior; LAT, Lateral

All elbow joint radiographs were acquired using digital radiography systems: an AXIOM Aristos unit Siemens, Forchheim, Germany, (1,448/2,643, 54.8%); a RADspeed Pro unit, Shimadzu, Kyoto, Japan (933/2,643, 35.3%); and a DX6290 unit, ANGELL, Guangzhou, China (262/2,643, 9.9%), ensuring complete anonymization of all sensitive information. Utilizing this dataset, we trained AI QC models, and the flowchart is illustrated in Fig. 1.

### Data annotations

To ensure that the image quality met diagnostic standards, a panel consisting of radiology residents, radiologists, and technicians conducted thorough discussions and evaluations referencing to the elbow joint radiograph QC standards [4], and ultimately identifying five critical quality control criteria to assess the effectiveness of our subsequent AI-based models. Specifically, three criteria were established for the AP view (AP elbow joint positioning, AP olecranon fossa positioning, and AP joint space) and two for the LAT view (LAT elbow joint positioning, and LAT flexion angle), as detailed below.

### QC standards for AP view

1. To minimize image distortion and ensure complete capture of joint structures, the image center should display the distal humerus, proximal ulna and radius, and their inter-joint space. This requires precise measurement of the elbow joint’s central position.
2. To ensure correct alignment of the elbow’s anatomical structure, the olecranon fossa should be centrally positioned between the medial and lateral epicondyles of the humerus, slightly biased toward the ulnar side. This involves measuring the distance from the olecranon fossa to the medial and lateral epicondyles.
3. To ensure proper joint alignment, the elbow joint space should be fully expanded and horizontally displayed. This requires measuring key points related to the joint space.

### QC standards for LAT view

1. To ensure the complete display of joint structures, the elbow joint should be centered in the image. This involves measuring the elbow joint’s central position.
2. To reflect normal anatomical alignment and provide a clear view of the joint space, the elbow’s flexion angle should be approximately 90°. This requires measuring the angle between the humerus and the radius in the LAT view.

Computing QC results directly from images poses significant challenges. We defined a set of key points aiming to delineate crucial anatomical landmarks in elbow joint images. YOLOv8 is an object detection algorithm that can predict both bounding box and key points. Accordingly, by integrating the described QC standards with the features of the YOLOv8 algorithm, we employed one target detection box and seven key points for AP view and one target detection box and three key points for LAT view. The “target detection box” refers to the bounding box used in object detection tasks for our YOLOv8 model. The primary function of the target detection box is to accurately locate the elbow joint’s boundaries, while the key points are designated to precisely mark specific anatomical locations. A comprehensive definition of the detection box and key points, manifested by their coordinates, is provided in Table 1.

**Table 1:** Detailed description of key points together with their coordinates

AP/LAT	Box/Key point	Description	Coordinates
AP	A	Box for detecting the location of the elbow joint in the AP view	X_A,_ Y_A_
	1	The key point of the medial epicondyle of the humerus	X_1,_ Y_1_
	2	The key point on the lateral epicondyle of the humerus	X_2,_ Y_2_
	3	The key point located in the medial joint space inferior to the humerus	X_3,_ Y_3_
	4	The key point located in the lateral joint space inferior to the humerus	X_4,_ Y_4_
	5	The key point located in the medial joint space superior to the radius	X_5,_ Y_5_
	6	The key point located in the lateral joint space superior to the radius	X_6,_ Y_6_
	7	The key point located at the central position of the coronoid fossa in the elbow joint	X_7,_ Y_7_
LAT	A	Box for detecting the location of the elbow joint in the LAT view	X_A,_ Y_A_
	1	The key point located at the mid-humerus	X_1,_ Y_1_
	2	The key point located at the center of the humeral trochlea	X_2,_ Y_2_
	3	The key point located at the mid-radius	X_3,_ Y_3_

*AP* Anteroposterior, *LAT* Lateral

**Fig. 2:**
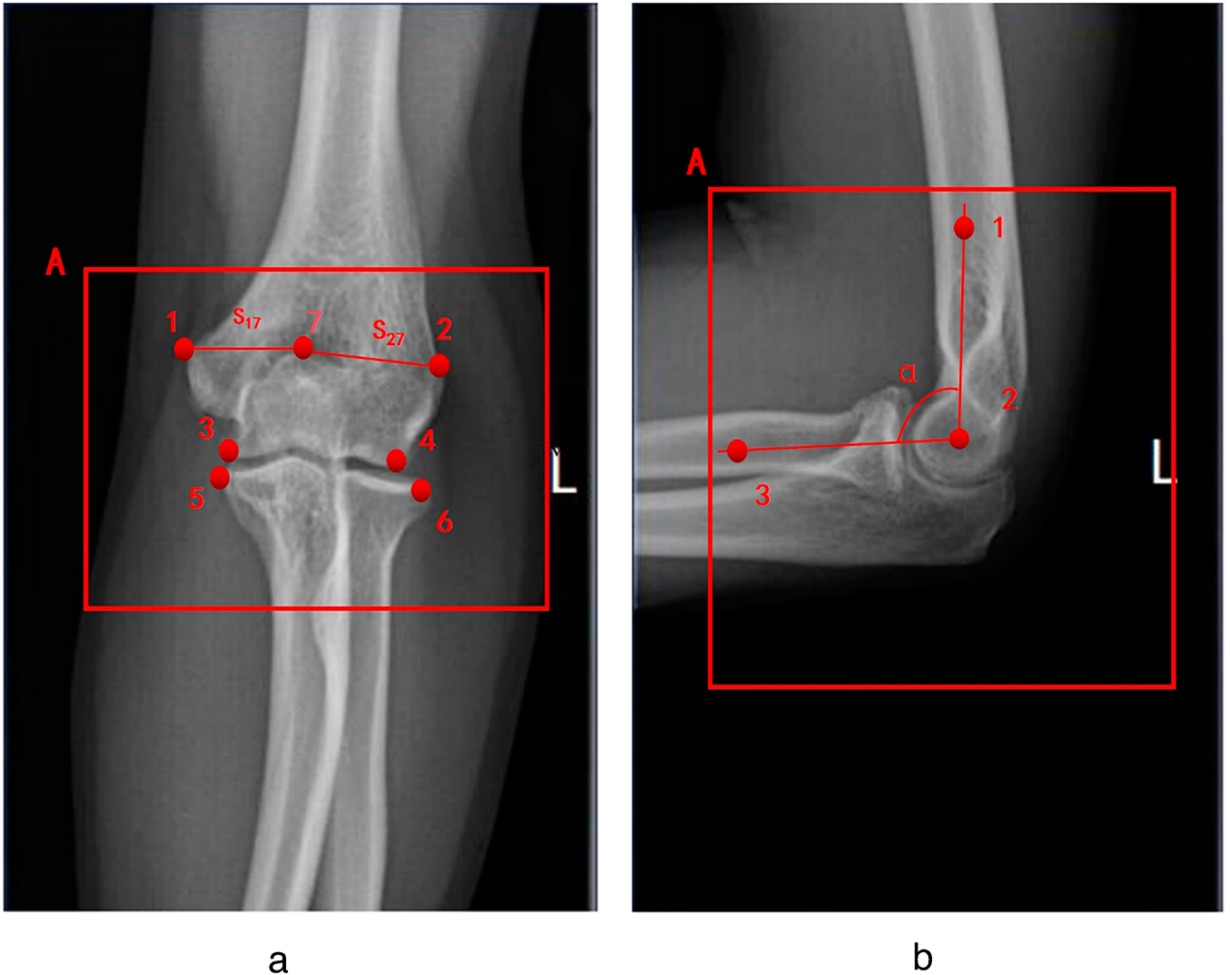
Annotations of key points and auxiliary lines. **a** Anterioposterior view of the elbow joint. **b** Lateral view of the elbow joint. Auxiliary lines S_17_ and S_27_ in the anterioposterior view represent distances between points 1 and 7, and between points 2 and 7, respectively, along with the flexion angle α in the lateral view are shown

To ensure the accuracy of data annotation, two radiology technicians (X.H. and W.C.), with 6 and 20 years of experience, respectively, initially annotated all elbow joint radiographs by utilizing Labelme software (version 5.3.1). Following this initial phase, a committee comprising two seasoned radiologists with 10 and 26 years of clinical experience, respectively, reviewed all annotations. They corrected any inaccuracies in the key points and discarded any ambiguous labels, ensuring that all annotations were consistent and undisputed. The final, verified annotations were confirmed to be error-free. Specific examples of the annotations are illustrated in Fig. 2.

### Image quality criteria and quantitative indices

Through the above-detailed standards and computational metrics, we conducted a comprehensive image quality assessment of elbow joint radiographs and also provided clear and definitive evaluation guidelines for subsequent deep learning models focusing on the key points detection tasks (Table 2). Based on our five criteria for elbow joint image quality assessment, we proposed two YOLOv8L-based detection models for the AP and LAT images of the elbow joint to automate QC assessment. We used the AP model to assess the predictive performance of one target detection box and seven key points in the AP view, whereas the LAT model was used to assess the predictive performance of three key points in the LAT view. Specifically, in the AP view, the primary role of the target detection box was to locate the position of the elbow joint. In the processing of LAT view, we used the key point located at the center of the humeral trochlea (key point 2) for precise localization of the elbow joint.

**Table 2:** Quality criteria and quantitative metrics for elbow joint radiographs

AP/LAT	Quality criteria	Description of criteria	Quantitative indices	Definition of quantitative indices measurement
AP	Positioning of elbow joint	The elbow joint should be centered in the image with an appropriate size.	X_A_, Y_A_	The center coordinates (X_A_, Y_A_) of Box A should be at the center of the normalized image.
	Positioning of olecranon fossa	The olecranon fossa should be centered between the medial and lateral epicondyles of the humerus, slightly toward the ulnar side.	${S}_{17}=\sqrt{{({x}_{1}-{x}_{7})}^{2}+{({y}_{1}-{y}_{7})}^{2}}$ ${S}_{27}=\sqrt{{({x}_{2}-{x}_{7})}^{2}+{({y}_{2}-{y}_{7})}^{2}}$	The distance S_17_ from key point 1 to key point 7 should be slightly greater than the distance S_27_ from key point 2 to key point 7.
	Joint space	The joint space should be fully open and displayed horizontally.	Y_3_,Y_4_,Y_5_,Y_6_	Y-coordinates of key points 3 and 4 should be greater than those of key points 5 and 6.
LAT	Positioning of elbow joint	The elbow joint should be centered in the image with an appropriate size.	X_2,_ Y_2_	Coordinates (X_2,_ Y_2_) of key point 2 should be within the central region of the normalized image.
	Flexion angle	The angle between the humerus and radius should be approximately 90°.	${{{\rm{\alpha }}}}={\cos }^{-1}\frac{(\overrightarrow{{{{{\rm{P}}}}}_{1}{{{{\rm{P}}}}}_{2}}{{{\rm{\cdot }}}}\overrightarrow{{{{{\rm{P}}}}}_{2}{{{{\rm{P}}}}}_{3}})}{{{{|}}}{{{{\rm{P}}}}}_{1}{{{{\rm{P}}}}}_{2}{{{|}}}\times {{{|}}}{{{{\rm{P}}}}}_{2}{{{{\rm{P}}}}}_{3}{{{|}}}}$	α between the line passing through key points 1 and 2 and the line passing through key points 2 and 3. In the normalized image coordinates, angle α should be close to 90°.

The vectors *P*_*1*_*P*_*2*_ and *P*_*2*_*P*_*3*_ denote the segments from points 1 to 2 and 2 to 3, while *|P*_*1*_*P*_*2*_*|* and *|P*_*2*_*P*_*3*_*|* represent the distances from points 1 to 2 and 2 to 3, respectively

*AP* Anteroposterior, *LAT* Lateral

### Preprocessing

We optimized a data preprocessing method for x-ray image analysis using Python-3.9.13. We utilized the pydicom library to read DICOM files and employed the PIL library to convert the DICOM into JPEG images instead of PNG format because the impact of JPEG compression on image quality is minimal while reducing storage space and processing requirements. Subsequently, the images were normalized and resized into 640 × 640 using the built-in functionality of YOLOv8. Additionally, we used the built-in data augmentation library provided by YOLOv8. The parameters for image augmentation were as follows: brightness adjustment (hsv-v = 0.4), translation (translate = 0.1), scaling (scale = 0.5), vertical flipping (flipud = 0.5), horizontal flipping (fliplr = 0.5), and image mosaic (mosaic = 1.0).

### Model training

We employed the YOLOv8 for two primary tasks in elbow joint x-ray analysis: detecting the elbow joint and localizing key anatomical points. The model delineated the joint’s location with a bounding box in AP and LAT views, facilitating accurate localization of key anatomical points. Seven key points in the AP view and three in the LAT view were identified for automated QC.

**Fig. 3:**
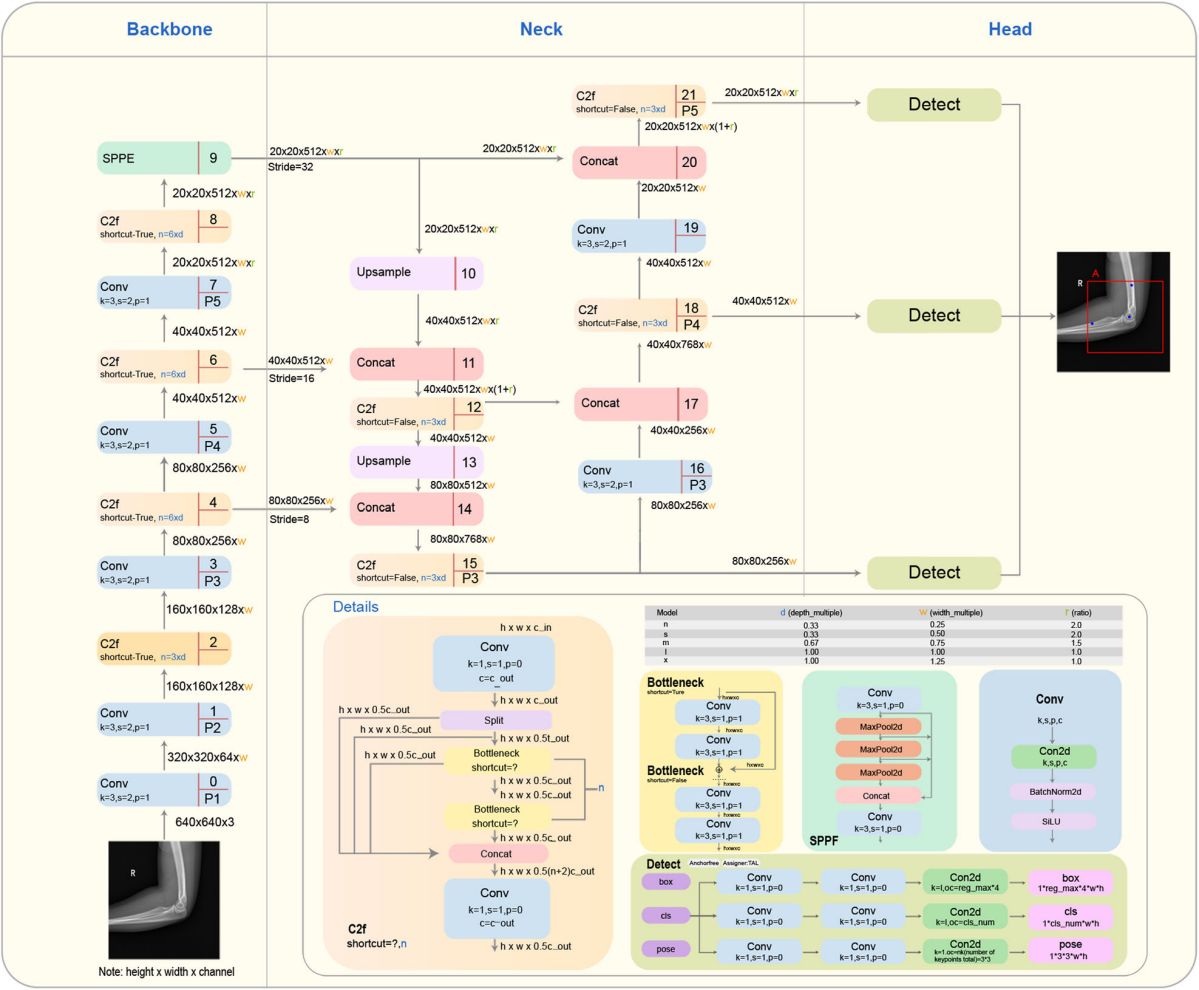
The architecture of the YOLOv8 algorithm, which is divided into three parts, including backbone, neck, and head

In this study, we selected the YOLOv8 Large (YOLOv8L) variant, the precise and extensive models in the YOLOv8 series, characterized by a parameter size of 43.61 million, to train our key points detection models for both AP and LAT views of the elbow joint, and the architecture of the YOLOv8 algorithm is shown in Fig. 3. The architecture consists of three key components: a backbone for feature extraction from elbow x-ray images, a neck for feature fusion, and a head for outputting the final predictions, including the classification of object detection box, the position and size of the detection box, and the position of key points. Transfer learning was employed using pre-trained weights from the YOLOv8L. The training regimen utilized the following software configurations: Ultralytics YOLOv8.0.62, Python-3.9.13, and PyTorch-1.13.1 on a GPU framework. The computational setup included a server-grade computer powered by an AMD Ryzen Threadripper 5975wx CPU and an NVIDIA GeForce RTX 3,090 GPU. Key training parameters were as follows: optimizer set to stochastic gradient descent (SGD), an initial learning rate of 0.001, and a training duration of 100 epochs.

We assessed our model’s object detection accuracy using standard metrics: mean average precision (mAP), precision, and recall. We evaluated bounding box accuracy based on intersection over union (IoU) and key point accuracy based on Object Keypoint Similarity (OKS), referencing the published research on YOLO-Pose [19]. The mAP was computed at 0.5 threshold (mAP50) for basic accuracy and at 0.5 to 0.95 incremental thresholds (mAP50–95) for detailed analysis.

### Assessment of models

This study utilized the intraclass correlation coefficient (ICC) to assess consistency between the AI system and clinical physician in annotating elbow joint on x-ray images in the test set. We used the ICC (3, k) model, which is a “two-way mixed effects, absolute agreement, and average measures” model, evaluating consistency between multiple raters by considering both subject and rater variability [20, 21]. We used this model to assess the absolute agreement between our AI system and clinical physicians, ensuring precise and clinically relevant consistency [22]. ICC above 0.75 indicated good reliability, and above 0.9, excellent reliability.

We evaluated the AI model’s correlation with clinical radiologists using key QC indicators in elbow joint x-ray imaging, including joint positioning in AP and LAT views, coronoid fossa location in AP, joint space in AP, and flexion angle in LAT. Through detailed analysis of the test set, we thoroughly evaluated the performance of the AI model in actual clinical applications.

### Clinical efficiency evaluation

The time efficiency of image quality control using the YOLOv8-QC model was compared with that of the traditional manual method on the test set. Two radiologists, Analyst 1 (X.D., 3 years of experience) and Analyst 2 (X.H., 6 years of experience), independently conducted the tests in two phases. The first phase involved traditional manual quality control, where the analysts manually evaluated the quality of elbow joint radiographs. The second phase utilized our AI-assisted quality control, employing the image quality assessment results automatically generated by the YOLOv8-QC model.

## Results

### Performance evaluation of models for AP and LAT views

**Fig. 4:**
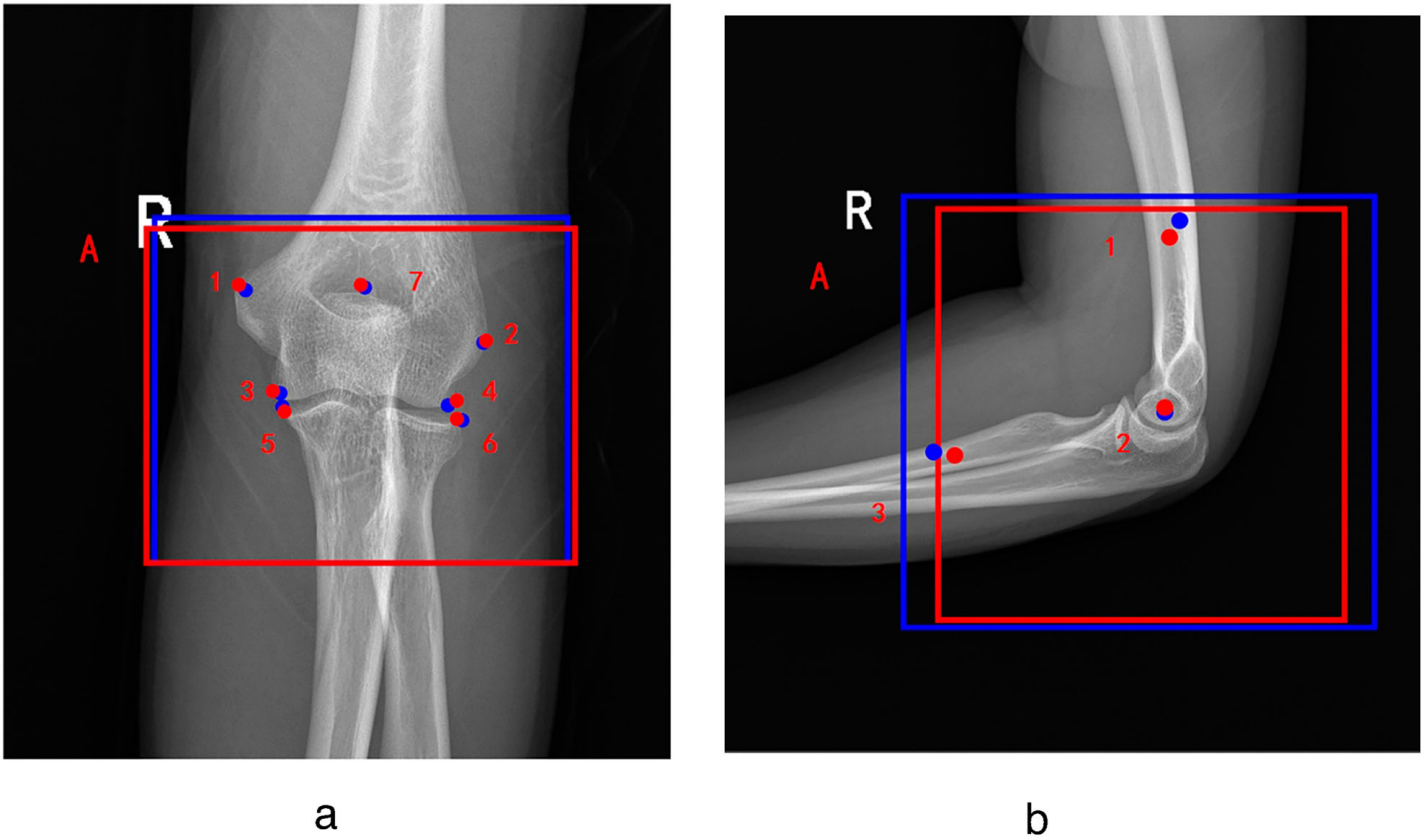
Visualization of the artificial intelligence (AI) models’ predictions and clinician’s annotation. The red key points and box are from the clinician’s annotations, and the blue key points and boxes are generated by the AI model. **a** Anterioposterior view, showing one target detection box and seven key points; **b** lateral view, showing one target detection box and three key points

After completing the training for the development of AP and LAT models, performance in the evaluation of the elbow joint x-ray image quality from both views is shown in Table 3. For identifying box and points using the AP model, precision reached more than 0.99, with recall rates of more than 0.98, mean mAP50 scores of more than 0.99, and mAP50-95 scores of more than 0.81 and more than 0.99, respectively. To identify key points using the LAT model, the precision was 0.993, with a recall rate of 0.994, mAP50 of 0.991, and mAP50-95 of 0.987. Examples of the key point detection results and the clinician’s annotations are visualized in Fig. 4, where the red key points and box are from the clinician’s annotations, and the blue key points and box are generated by the AI models.

**Table 3:** Performance in AP and LAT models

	Precision	Recall	mAP50	mAP50-95
AP (box)	0.988	0.984	0.995	0.814
AP (points)	0.995	0.996	0.994	0.992
LAT (points)	0.993	0.994	0.991	0.987

*AP* Anteroposterior, *LAT* Lateral

### Image quality assessment by AI model *versus* radiologists

In assessing the ICC in AP and LAT projections of elbow joint radiography between the AI-based model and clinical doctors, our findings demonstrated high consistency across most evaluation standards (Table 4). For AP, the elbow joint positioning showed excellent consistency with an ICC of more than 0.98, and the positioning of the coronoid fossa also exhibited strong consistency with an ICC of more than 0.95. The assessment of the joint space further reinforced these findings, showing almost perfect consistency with an ICC of more than 0.95. In LAT, the elbow joint positioning exhibited high consistency with an ICC of more than 0.98. However, the assessment of the flexion angle showed slightly lower but still considerable consistency with an ICC of 0.865.

**Table 4:** ICC measurements between the AI-based model and clinicians in AP and LAT views

Projection direction	Quality criteria	Quantitative indices	ICC (95% CI)
AP	Positioning: elbow joint	X_A_	0.987 (0.98–0.99)
		Y_A_	0.991 (0.99–0.99)
	Positioning: olecranon fossa	S_17_	0.964 (0.95–0.97)
		S_27_	0.951 (0.94–0.96)
	Joint space evaluation	Y_3_	0.998 (0.99–1.0)
		Y_4_	0.997 (0.99–1.0)
		Y_5_	0.998 (0.99–1.0)
		Y_6_	0.959 (0.95–0.97)
LAT	Positioning: elbow joint	X_2_	0.994 (0.99–1.0)
		Y_2_	0.986 (0.98–0.99)
	Flexion angle evaluation	α	0.865 (0.83–0.95)

*AP* Anteroposterior, *CI* Confidence interval, *ICC* Intraclass correlation coefficient, *LAT* Lateral

**Fig. 5:**
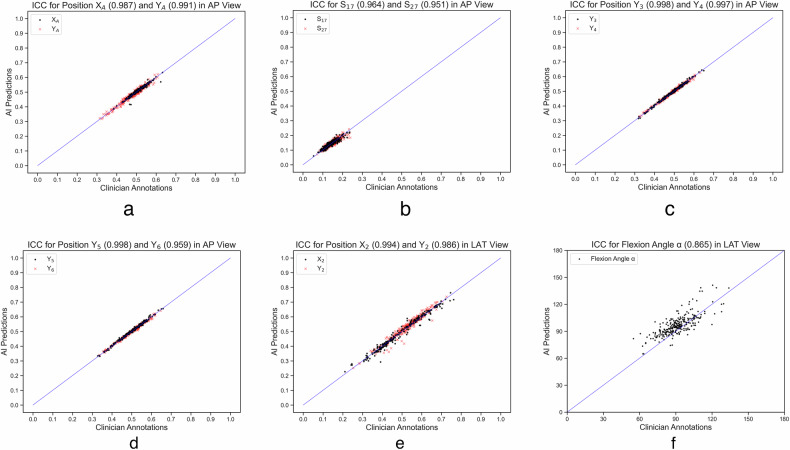
Scatter plots of correlations between AI predictions and clinician annotations in the test set. **a** ICC for X_A_ and Y_A_ in AP view; **b** ICC for S_17_ and S_27_ in AP view; **c** ICC for Y_3_ and Y_4_ in AP view; **d** ICC for Y_5_ and Y_6_ in AP view; **e** ICC for X_2_ and Y_2_ in LAT view; **f** ICC for flexion angle α in LAT view. AP, Anteroposterior; ICC, Intraclass correlation coefficient; LAT Lateral

The correlation between the AI-based model and clinical radiologists across multiple key QC indicators in elbow joint x-ray imaging is depicted in Fig. 5. In the figure, the blue diagonal line represents perfect consistency between the AI model and clinical radiologist assessments. The axes in Fig. 5a–e are normalized, while the flexion angle range in Fig. 5f is set from 0 to 180°. Figure 5a–e presents scatter plots for AP elbow joint positioning, AP coronoid fossa location, AP joint space, and LAT elbow joint positioning, respectively. In these charts, most data points are tightly clustered near the blue diagonal line, representing perfect consistency, demonstrating the high consistency and minimal deviation of the AI model’s assessments compared to those of clinical radiologists in these QC indicators. Figure 5f specifically focuses on the scatter distribution of the LAT flexion angle.

### Clinical efficiency

**Fig. 6:**
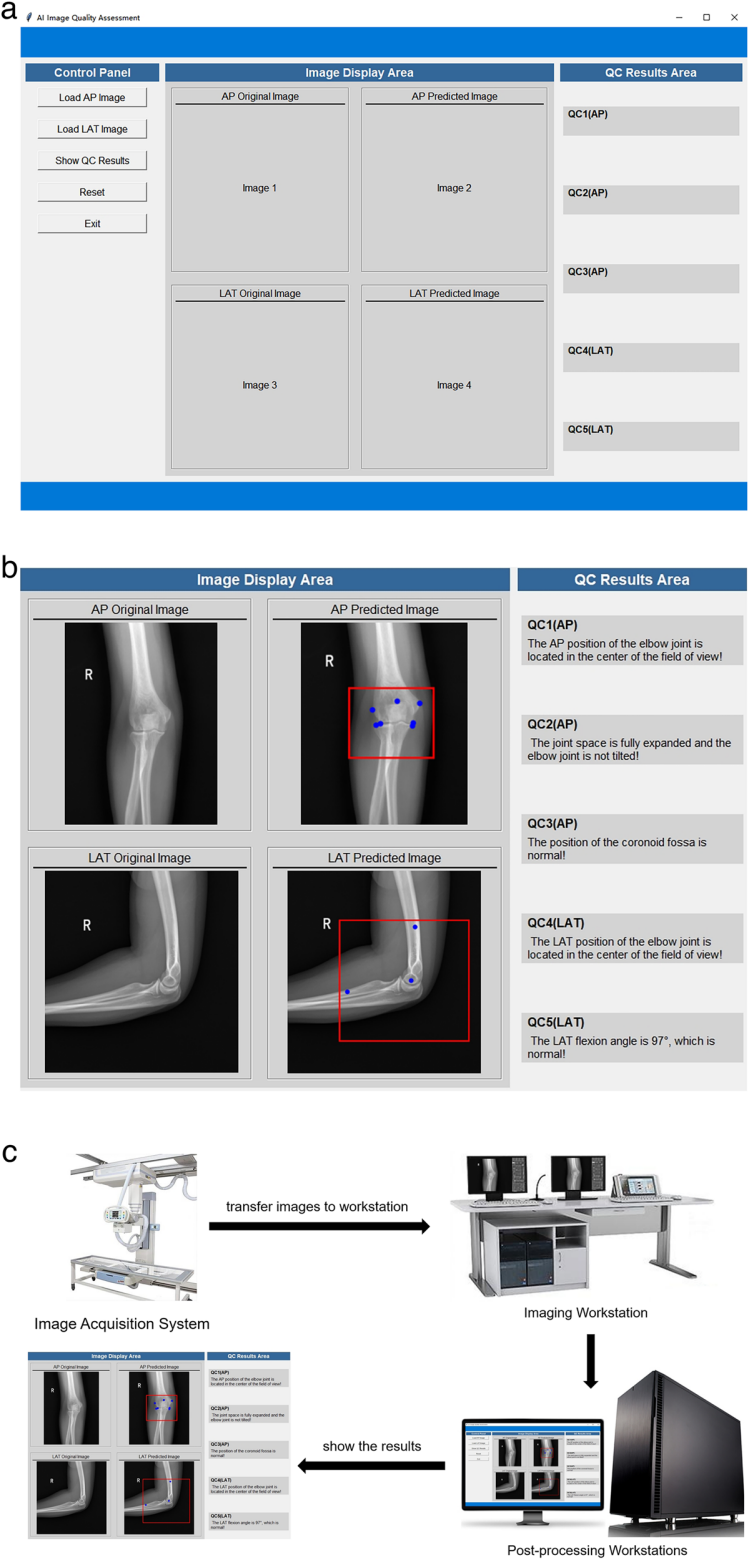
YOLOv8-QC GUI software and its application. **a** YOLOv8-QC GUI software. **b** QC results available to users. **c** Application of YOLOv8-QC GUI software. AP, Anteroposterior; LAT, Lateral; QC, Quality control; GUI, Graphical user interface

This study developed a graphical user interface (GUI) software package using Python’s Tkinter module (Fig. 6a) based on our YOLOv8-QC model, which allowed for both individual and simultaneous quality assessments of AP and LAT images. The software yielded QC results available to users (Fig. 6b). Two analysts independently evaluated a test set comprising 260 AP images and 263 LAT images. In the initial phase of traditional manual quality control, Analyst 1 took 165 min to evaluate AP images and 131 min for LAT images, while Analyst 2 took 151 min to evaluate AP images and 123 min for LAT images. In the second phase, with the AI-assisted quality control, the evaluation time for Analyst 1 reduced to 99 min for AP images and 75 min for LAT images, and the evaluation time for Analyst 2 reduced to 82 min for AP images and 66 min for LAT images. Under the AI assistance, the average processing time for AP and LAT images was 91 min for AP images and 70 min for LAT images. The results demonstrated that our AI assistance significantly reduced the time for image quality control, with reductions of 42.59% for AP images and 44.63% for LAT images (*t*-test, *p* < 0.001). Consequently, the GUI software was implemented on our post-processing workstations associated with DR systems, employing the procedural framework depicted in Fig. 6c.

We installed the GUI software on the post-processing workstations of the DR system. This YOLOv8-QC GUI enabled real-time assessment of elbow joint images captured by radiographers. When substandard images were detected, the radiographers could re-photograph these views.

## Discussion

We proposed an innovative AI-driven automatic QC model for elbow joint x-ray images. This model employs the cutting-edge YOLOv8 algorithm to identify predefined target detection box and key points within the images. We use this algorithm to train the model for the first time in the field of image quality control. Computing QC results directly from images poses significant challenges [10], so the models employ a series of meticulous geometric calculations to transform these detection boxes and key points into five above-described QC standards.

Our results indicate that the AI-based models are reliable across all five quality control standards. For the ICC assessment in the test set, the proposed AI model and clinical doctors showed high consistency in evaluating the AP elbow joint positioning coordinates X_A_ and Y_A_. In assessing the AP olecranon fossa positioning distance parameters S_17_ and S_27_, the ICC values showed a high correlation of our AI models with radiologists. For the four key points of the AP joint space (Y_3_, Y_4_, Y_5_, Y_6_), the ICC values were excellent. In the LAT assessments, the ICC values for the elbow joint positioning coordinates X_2_ and Y_2_ were also excellent, and the ICC value for the LAT elbow flexion angle was good. Compared to other QC indicators, the scatter distribution in this chart is relatively more dispersed, reflecting the specific challenges in measuring the LAT flexion angle. This dispersion may stem from the complexity of measuring flexion angle, such as minor deviations in the AI model’s predictions of LAT flexion angle. Nevertheless, most data points are still closely clustered near the blue diagonal line, representing perfect consistency, indicating the high accuracy and reliability of the AI model in measuring the LAT flexion angle. In summary, the results showed high consistency and reliability of our AI model in elbow joint x-ray image QC, providing strong support for its application in clinical practice.

Furthermore, based on the trained model, we developed a GUI software named YOLOv8-QC. This software is specifically designed to automatically assess the quality of AP and LAT x-ray images of the elbow joint and to display the assessment results in real time. The results demonstrated that our AI assistance significantly reduced the time for image quality control, with reductions of 42.6% for AP images and 44.6% for LAT images. Compared to the conventional manual quality control method, the GUI software enhances the efficiency in quality management, presenting practical implications. This not only significantly reduces the time required for image quality assessment in clinical settings, but also optimizes workflow in radiology departments, enhancing overall efficiency.

Several AI models in different anatomical regions have been published and can provide timely feedback on unqualified images [9–12, 23]. Nousiainen et al [23] employed convolutional neural networks for automated diagnostic quality control of chest radiographs, achieving immediate feedback. Similarly, Yu et al [9] developed a quality control system combining deep learning models with linear regression to assess the layout and positioning of chest radiographs with high accuracy. In the context of knee digital radiography, Sun et al [10] utilized a key inspection algorithm for quality control, achieving high concordance with clinical experts in evaluating image quality issues caused by incorrect positioning. Mairhofer et al [11] developed a modular deep learning framework leveraging anatomical features for automated quality control of ankle radiographs, achieving an average accuracy of 94.1%, surpassing radiologists’ performance. Chen et al [12] developed a U-Net-based framework for quality control of lumbar spine x-rays, accurately segmenting and identifying unqualified images. Our study, utilizing advanced object detection algorithms, successfully automated the quality assessment of elbow digital radiographs, thereby addressing a critical gap in AI-based quality control for this anatomical region and broadening the potential applications of AI in medical imaging quality assessment.

Although our model demonstrates commendable performance in its current application, certain limitations warrant attention. As an initial feasibility study, we have trained the model using only normal elbow radiographs, and the model’s performance in extreme scenarios, such as severe elbow deformities or rare pathological conditions, remains to be further validated. Future research will consider training AI quality control models for more complex elbow conditions, including fractures, metallic artifacts, and postoperative changes. Additionally, our study currently focuses on the five QC criteria for image positioning. We aim to explore additional quantitative metrics in the future, including imaging quality and supplementary positioning standards, to propose a more comprehensive and clinically applicable QC system. Finally, we plan to evaluate other quantitative metrics to assess the consistency between clinical practitioners and the AI model.

In conclusion, the practical application prospects of the findings in clinical practice are promising. By integrating AI-based automatic quality control technology for elbow x-ray images, we can effectively address the issues associated with traditional manual quality control, such as high workload and subjective inconsistency in evaluation results. This technology enables real-time monitoring of imaging quality, significantly reducing the potential for errors by radiographic technicians during the imaging process, thereby effectively preventing disputes between medical practitioners and patients. The automated quality assessment can further ensure a high degree of consistency and accuracy in diagnostic images, and enhance the quality of medical services provided to patients by optimizing the medical process. Furthermore, with the continuous advancement of deep learning technology in the field of medical imaging, our models have the potential to become an important component of future intelligent healthcare systems.

## Data Availability

Please contact the corresponding author (TWC) for data requests.

## Abbreviations

AI: Artificial Intelligence
AP: Anteroposterior
GUI: Graphical user interface
ICC: Intraclass correlation coefficient
LAT: Lateral
mAP: Mean average precision
QC: Quality control
YOLOv8: “You Only Look Once” version 8

## References

[1] Javed M, Mustafa S, Boyle S, Scott F (2015) Elbow pain: a guide to assessment and management in primary care. Br J Gen Pract 65:610–612. 10.3399/bjgp15X687625 26500317 10.3399/bjgp15X687625 PMC4617264

[2] Luceri F, Cucchi D, Rosagrata E et al (2021) Novel radiographic indexes for elbow stability assessment: part A—cadaveric validation. Indian J Orthop 55:336–346. 10.1007/s43465-021-00407-4 34306546 10.1007/s43465-021-00407-4 PMC8275710

[3] Sheehan SE, Dyer GS, Sodickson AD, Patel KI, Khurana B (2013) Traumatic elbow injuries: what the orthopedic surgeon wants to know. Radiographics 33:869–888. 10.1148/rg.333125176 23674780 10.1148/rg.333125176

[4] Crosby NE, Greenberg JA (2014) Radiographic evaluation of the elbow. J Hand Surg Am 39:1408–1414. 10.1016/j.jhsa.2014.04.035 24888528 10.1016/j.jhsa.2014.04.035

[5] Jones AK, Polman R, Willis CE, Shepard SJ (2011) One year’s results from a server-based system for performing reject analysis and exposure analysis in computed radiography. J Digit Imaging 24:243–255. 10.1007/s10278-009-9236-2 19885636 10.1007/s10278-009-9236-2 PMC3056967

[6] Huda W, Abrahams RB (2015) Radiographic techniques, contrast, and noise in X-ray imaging. AJR Am J Roentgenol 204:W126–W131. 10.2214/AJR.14.13116 25615772 10.2214/AJR.14.13116

[7] Huda W, Abrahams RB (2015) X-ray-based medical imaging and resolution. AJR Am J Roentgenol 204:W393–W397. 10.2214/AJR.14.13126 25794088 10.2214/AJR.14.13126

[8] Seiber K, Gupta R, McGarry MH, Safran MR, Lee TQ (2009) The role of the elbow musculature, forearm rotation, and elbow flexion in elbow stability: an in vitro study. J Shoulder Elb Surg 18:260–268. 10.1016/j.jse.2008.08.004 10.1016/j.jse.2008.08.004 19046641

[9] Meng Y, Ruan J, Yang B et al (2022) Automated quality assessment of chest radiographs based on deep learning and linear regression cascade algorithms. Eur Radiol 32:7680–7690. 10.1007/s00330-022-08771-x 35420306 10.1007/s00330-022-08771-x

[10] Sun H, Wang W, He F et al (2023) An AI-based image quality control framework for knee radiographs. J Digit Imaging 36:2278–2289. 10.1007/s10278-023-00853-6 37268840 10.1007/s10278-023-00853-6 PMC10501977

[11] Mairhöfer D, Laufer M, Simon PM et al (2021) An AI-based framework for diagnostic quality assessment of ankle radiographs. PMLR 143:484–496

[12] Chen X, Deng Q, Wang Q et al (2022) Image quality control in lumbar spine radiography using enhanced U-net neural networks. Front Public Health 10:891766. 10.3389/fpubh.2022.891766 35558524 10.3389/fpubh.2022.891766 PMC9087032

[13] Wang Z, Liu Y, Duan S, Pan H (2023) An efficient detection of non-standard miner behavior using improved YOLOv8. Comput Electr Eng 112:109021. 10.1016/j.compeleceng.2023.109021

[14] Inui A, Mifune Y, Nishimoto H et al (2023) Detection of elbow OCD in the ultrasound image by artificial intelligence using YOLOv8. Appl Sci 13:7623. 10.3390/app13137623

[15] Ju RY, Cai W (2023) Fracture detection in pediatric wrist trauma x-ray images using YOLOv8 algorithm. Sci Rep 13:20077. 10.1038/s41598-023-47460-7 37973984 10.1038/s41598-023-47460-7 PMC10654405

[16] Sharma N, Baral S, Paing MP, Chawuthai R (2023) Parking time violation tracking using YOLOv8 and tracking algorithms. Sensors 23:5843. 10.3390/s23135843 37447693 10.3390/s23135843 PMC10346361

[17] Chabi Adjobo E, Sanda Mahama AT, Gouton P, Tossa J (2023) Automatic localization of five relevant dermoscopic structures based on YOLOv8 for diagnosis improvement. J Imaging 9:148. 10.3390/jimaging9070148 37504825 10.3390/jimaging9070148 PMC10381143

[18] Li P, Zheng J, Li P, Long H, Li M, Gao L (2023) Tomato maturity detection and counting model based on MHSA-YOLOv8. Sensors 23:6701. 10.3390/s23156701 37571485 10.3390/s23156701 PMC10422388

[19] Maji D, Nagori S, Mathew M, Poddar D (2022) YOLO-pose: enhancing YOLO for multi person pose estimation using object keypoint similarity loss. In: Proceedings of the IEEE/CVF Conference on Computer Vision and Pattern Recognition, New Orleans. pp 2637–2646. 10.1109/CVPRW56347.2022.00297

[20] Shrout PE, Fleiss JL (1979) Intraclass correlations: uses in assessing rater reliability. Psychol Bull 86:420. 10.1037/0033-2909.86.2.420 18839484 10.1037//0033-2909.86.2.420

[21] McGraw KO, Wong SP (1996) Forming inferences about some intraclass correlation coefficients. Psychol Methods 1:30. 10.1037/1082-989X.1.1.30

[22] Koo TK, Li MY (2016) A guideline of selecting and reporting intraclass correlation coefficients for reliability research. J Chiropr Med 15:155–163. 10.1016/j.jcm.2016.02.012 27330520 10.1016/j.jcm.2016.02.012 PMC4913118

[23] Nousiainen K, Mäkelä T, Piilonen A, Peltonen JI (2021) Automating chest radiograph imaging quality control. Phys Med 83:138–145. 10.1016/j.ejmp.2021.03.014 33770747 10.1016/j.ejmp.2021.03.014

